# Spatial extreme value analysis to project extremes of large-scale indicators for severe weather

**DOI:** 10.1002/env.2234

**Published:** 2013-09-18

**Authors:** Eric Gilleland, Barbara G Brown, Caspar M Ammann

**Affiliations:** a Research Applications Laboratory, National Center for Atmospheric ResearchP.O. Box 3000, Boulder, CO 80307, U.S.A.

**Keywords:** severe storms, reanalysis data, conditional extreme value modeling, river flow

## Abstract

Concurrently high values of the maximum potential wind speed of updrafts (*W*_max_) and 0–6 km wind shear (Shear) have been found to represent conducive environments for severe weather, which subsequently provides a way to study severe weather in future climates. Here, we employ a model for the product of these variables (WmSh) from the National Center for Atmospheric Research/United States National Center for Environmental Prediction reanalysis over North America conditioned on their having extreme energy in the spatial field in order to project the predominant spatial patterns of WmSh. The approach is based on the Heffernan and Tawn conditional extreme value model. Results suggest that this technique estimates the spatial behavior of WmSh well, which allows for exploring possible changes in the patterns over time. While the model enables a method for inferring the uncertainty in the patterns, such analysis is difficult with the currently available inference approach. A variation of the method is also explored to investigate how this type of model might be used to qualitatively understand how the spatial patterns of WmSh correspond to extreme river flow events. A case study for river flows from three rivers in northwestern Tennessee is studied, and it is found that advection of WmSh from the Gulf of Mexico prevails while elsewhere, WmSh is generally very low during such extreme events. © 2013 The Authors. Environmetrics published by JohnWiley & Sons, Ltd.

## 1. INTRODUCTION

Extreme weather events can cause substantial damage in terms of human lives, financial losses, loss of infrastructure necessary for society, etc. As climate changes, it is important to understand how extreme weather events may change as a result. Unfortunately, many of these phenomena occur at scales too fine to be properly resolved by climate models (e.g., severe thunderstorms, tornados, high winds). To more accurately analyze and project these phenomena, different methods have been proposed. Examples include (i) the use of statistical extreme value analysis (EVA) to project extremes of observed data or climate model simulations, possibly with covariates in the parameters, to account for changes in the process over time (e.g., Kharin and Zwiers, 2000, 2005; Fowler and Kilsby, 2003; Fowler *et al.*, 2005, 2007; Kharin *et al.*, 2007; Frei *et al.*, 2006; Fowler and Ekström, 2009); (ii) addressing connections between extremes and variables that are output by climate models and possibly downscaling from there (e.g., Benestad *et al.*, 2012); and (iii) utilizing large-scale indicators of severe weather that can be resolved by climate models (e.g., Frich *et al.*, 2002; Brooks *et al.*, 2003; Marsh *et al.*, 2007, 2009; Trapp *et al.*, 2007, 2009; Heaton *et al.*, 2011). Most of these large-scale indicator studies focused their attention on frequency of occurrence of severe weather environments and study averages and variability of such “proxy” events. An exception is Heaton *et al.* (2011), where return level maps obtained from a Bayesian Hierarchical Model applied to extreme value distributions are fit to reanalysis data separately at each grid point. The main objective for the present work is to introduce a modeling approach to describe the predominant spatial patterns of an indicator variable for severe storm environments conditional on the presence of extreme energy in the field in order to make projections into the future about these patterns, as well as to investigate how they may have changed over time. Our approach differs from that of Heaton *et al.* (2011) because we study *fields* of extremes, where it is possible for many grid points to not exhibit extreme behavior.

**Figure 1:**
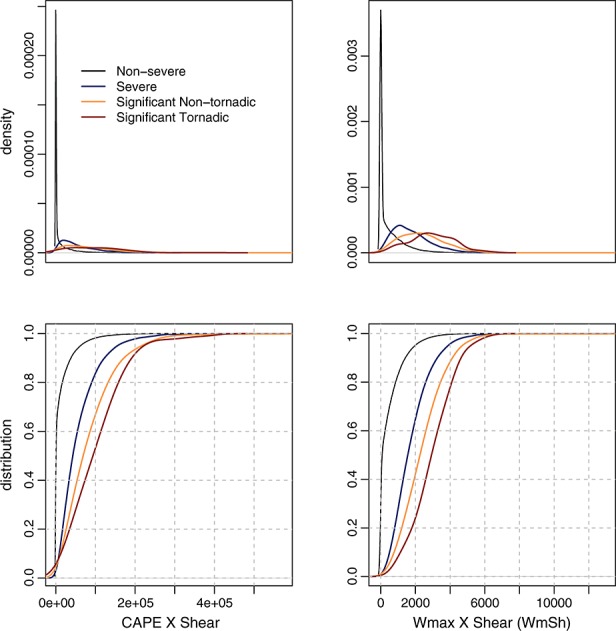
Probability density (top row) and cumulative distribution (bottom row) graphs for convective available potential energy × Shear (left column, J ⋅ m (kg ⋅ s) ^− 1^) and *W*_max_ × Shear (right column, m ^2^ s ^− 2^) conditional on storm categories as defined in Table 1. This figure is available in color online at http://wileyonlinelibrary.com/journal/environmetrics

Previous studies have found that concurrent high values of convective available potential energy (CAPE, J kg ^− 1^) and 0–6 km wind shear (Shear, m s ^− 1^; wind direction change across the lowest 6 km of the atmosphere) are associated with environments conducive to severe weather (see, e.g., Brooks *et al.*, 2003, 2007; Marsh *et al.*, 2007, 2009; Trapp *et al.*, 2009 for a summary). For example, Figure 1 shows probability distribution functions (dfs) for the product of CAPE and Shear (left panels) conditional on the storm categories described in Table 1. Separation can clearly be seen, particularly for the cumulative dfs (bottom left), where they shift toward larger values of the product as the storm category increases in severity. A transformation of CAPE to the maximum potential wind speed of updrafts, *W*_max_, with 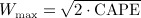 (cf. Holton, 2004; Trapp *et al.*, 2009), yields a variable in the same units as Shear (i.e., m s ^− 1^), and it has been suggested that concurrently high values of *W*_max_ and Shear are a better indicator of severe weather (personal communication from Harold E. Brooks, 2008). The right panels of Figure 1 confirm that the delineation of storm type by this transformation is clearer, if only slightly.

**Table 1:** Storm category definitions used in Figure 1

Non-severe	Hail < 1.9 cm (3/4 in) diameter
	Winds < 55 kts ( ≍ 28.29 m s ^− 1^) no tornado
Severe	Hail 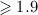 cm diameter
	Winds 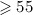 kts ( ≍ 28.29 m s ^− 1^) and < 65 kts ( ≍ 33.44 m s ^− 1^) or tornado
Significant	Hail 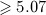 cm (2 in) diameter
non-tornadic	Winds 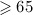 kts ( ≍ 33.44 m s ^− 1^)
Significant	Same as significant non − tornadic with F2 (or greater) tornado.
tornadic	

High values of CAPE, or its transform *W*_max_, are a necessary, but not sufficient, condition for severe weather. But when coinciding high Shear is present, the likelihood for severe storms increases rapidly. Therefore, severe weather is distributed across space following the combination of high *W*_max_ and high Shear. In addition to studying the product, it is informative to investigate each of these variables individually, particularly when considering changes in the future and how they are associated with different processes. However, we do not undertake this latter objective in the present study.

Based on their broad occurrence, *W*_max_ and Shear are often small and have strong spatial dependencies. Regions with concurrent large values of *W*_max_ and Shear play an important role in terms of whether or not severe storms will form. Whether or not such storms will have a large impact on lives, infrastructure and the environment will depend on their location (e.g., the recent 2012 Hurricane Sandy had a major impact because of where it came ashore). Therefore, it is not only important to determine the values of, for example, return levels of the indicators in a particular location (as, e.g., in Heaton *et al.,* 2011) but also to investigate their spatial patterns when extreme conditions exist over some subregion in the field. For example, spring is associated with high values of WmSh over the central USA where tornados are common at this time of year. It is important to determine if this region of intense WmSh (and tornadic activity) is becoming more (or less) intense and, whether or not it might be shifting in space, or becoming more (or less) widespread. Such a determination is particularly of interest when trying to establish risks associated with large-scale outbreaks of severe weather when emergency services are stretched to their limits. The modeling approach we introduce here is an important step in this direction, although such a complete analysis is beyond the scope of the present work.

Inferring extreme behavior for random variables in a spatial setting is still an active area of research (see, e.g., Yee and Wild, 1996; Gilleland and Nychka, 2005; Gilleland *et al.*, 2006; Cooley *et al.*, 2006, 2007; Fawcett and Walshaw, 2006; Huerta and Sansó, 2007; Yee and Stephenson, 2007; Buishand and Zhou, 2008; Zhang *et al.*, 2008; Eastoe, 2009; Eastoe and Tawn, 2009; Ribatet, 2009; Smith and Stephenson, 2009; Cooley and Sain, 2010; Mendes *et al.*, 2010; Padoan *et al.*, 2010; Sang and Gelfand, 2010; Turkman *et al.*, 2010; Wang and Stoev, 2010; Davison *et al.*, 2012; Genton *et al.*, 2011; Reich and Shaby, 2012; Ribatet *et al.*, 2012; Fuentes *et al.*, 2013). The present study differs from these previous works in that, here, we characterize the spatial nature of a variable (WmSh) conditioned on its exhibiting extreme conditions somewhere in the field. Despite the fact that large regions may not experience extreme conditions at all, overall, a summary measure across the field is used to recognize that at least one or more regions experience particularly high values. Because these are large-scale variables available on a grid everywhere in the region, we are not concerned with spatial interpolation.

To this end, we employ the conditional extreme value model introduced by Heffernan and Tawn (2004), which allows for modeling the distribution function of one or more variables, conditional on another variable's being extreme. They showed that for a wide class of extreme dependence structures, the joint df, conditional on one variable's exceeding a high threshold, results in a dependence model with a specific structure. When modeling several variables conditional on the same variable, simultaneously, any dependence among these variables is also established.

To identify extreme conditions, we consider the overall field energy, rather than focusing on extreme values at individual locations. The approach is to condition on a univariate variable that measures the overall spatial energy of the processes at each point in time. There are many choices for such a measure, such as the field mean, sum, or a specific quantile. Several such measures were investigated, and here, we report our findings for the upper quartile, q75, as our measure of choice, which consistently yields physically meaningful results. In this way, it is possible to model the spatial distribution of the large-scale indicators given an extreme amount of spatial energy in the field regardless of whether every grid point in space is extreme or not. The objective is focused on the description of the field overall, its typical spatial coherence, relation to large-scale processes, and ultimately how variability and eventually future change in the large scale influences the spatio-temporal variability in severe weather indicators at finer scales.

A variation of the previous approach is also explored. In particular, WmSh is conditioned on extreme river flow from three rivers in northwestern Tennessee in order to qualitatively study the spatial patterns of WmSh when river flows become dangerously high. Results show that advection of WmSh from the Gulf of Mexico prevails, and elsewhere, WmSh tends to be very low. A further analysis (not described here) investigated river flow conditioned on extreme values of WmSh at nearby grid cells. The association was found to be positive but weak.

## 2. STATISTICAL METHODS

### 2.1. Extreme value analysis

Univariate EVA is concerned with the distribution of intense events that have a very low probability of occurrence. Theoretical results provide justification for using the generalized extreme value df for modeling maxima of a process taken over long blocks (e.g., annual maxima from a daily series available over many years) or equivalently for using the generalized Pareto (GP) df for modeling excesses over a high threshold. A Poisson process characterization for extreme events ties both of these approaches together and provides a mechanism for studying both the frequency and intensity of extreme events. It is possible to determine sources of variability for extremes of a random variable through covariate modeling within the parameters of the extreme value distributions (e.g., Coles, 2001; Gilleland and Katz, 2011). Incorporating covariates in this manner addresses how the distribution of extremes of one variable varies according to the covariates, be they extreme or not, and statistical tests, such as the likelihood ratio test, are available to test the significance of inclusion of the covariate(s) of interest in a similar manner as is performed in linear regression (see, e.g., Coles, 2001; Beirlant *et al.*, 2004; de Haan Ferreira, 2006; Reiss and Thomas, 2007, for a thorough treatment of EVA).

Of interest here is the threshold excess formulation


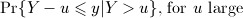
(1)

Heuristically, the previous probability is approximately equivalent to the GP df for a sufficiently large threshold, *u* (assuming it is non-degenerate in the limit), which is given by


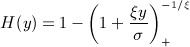


for − ∞ < *ξ* < ∞ (shape parameter), *σ* > 0 (scale parameter) and *z*_+_ = max(*z*,0). In particular, it is 1 − *H*(*y*) = Pr{*Y* − *u* > *y* | *Y* > *u*}that is of primary concern, and note that the limit as *ξ* tends to zero yields the exponential df, exp( − *y* / *σ*). For the present work, we will assume that *Y* follows a standard exponential df (i.e., exp( − *y*)) without loss of generality because it can be obtained through a simple transformation.

### 2.2. Conditional extreme value analysis

Before discussing the conditional model, it is helpful to review the copula dependence model formulation, which models the dependence of random variables on a common marginal df. That is,


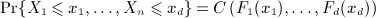


with *F*_*i*_ the marginal df for *X*_*i*_, *C* a unique function if the margins are continuous, called the copula, which describes the dependence of **X** = (*X*_1_, …, *X*_*d*_).

The conditional model framework employed here is that introduced by Heffernan and Tawn 2004) (hereafter, HT2004). We begin with the formulation used by (Heffernan and Resnick, 2007), which assumes that there are normalizing functions *a*(*Y* ) and *b*(*Y* ) such that for *y* > 0,


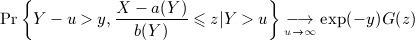
(2)

where *G* is a non-degenerate df. HT2004 found that, for a wide class of copula dependence models, using Gumbel margins for *X* and *Y*, that the forms for *a*(*Y* ) and *b*(*Y* ) fell into the simple class


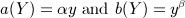
(3)

when *X* and *Y* are positively associated, where *α* ∈ [0,1] and *β* ∈ ( − ∞, 1). For negatively associated *X* and *Y*, another more complicated form was found; however, here, we follow Keef *et al*. (2013a, 2013b), who use a Laplace transformation, which ensures Equation (3) is valid for both positively and negatively associated random variables, where now *α* ∈ [ − 1,1]. The parameters *α* and *β* control the dependence between the variables. Because these two parameters are not independent of each other, interpretation of their values is not straightforward. Heuristically, using the Laplace transformation, *α* < 0 implies negative dependence, and *α* > 0 implies positive dependence, with weak dependence if *α* is close to zero. The parameter *β* measures the variability of the dependence with highly negative values indicating lower variability. As a reviewer pointed out, however, it is possible for *α* to be zero even when strong dependence exists (see HT2004 for an example).

Allowing *Z* = (*X* − *a*(*Y* )) / *b*(*Y* ), it is easy to see that Equation (2) implies conditional independence between *Z* and *Y* given *Y* > *u*, so that the first part of the product on the RHS of Equation (2) results directly from 1 − *H*(*y*) for the standardized exponential case assumed here. No simple closed-form expression exists for *G*. From Equations (2) and (3), we have that


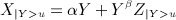
(4)

where the subscript on *X* and *Z* is there to emphasize the condition on *Y* > *u* with *u* large.

Subsequently, all that is needed in order to estimate the joint df of *X* and *Y*, conditional on *Y* > *u* for *u* large, is to know the parameters *α* and *β* and the df *G*. Estimation for the parameters *α* and *β* is an active area of research (see, e.g., Keef *et al.*, 2009a, 2009b, 2013a, 2013b), and estimation of *G* can be performed through resampling from the empirical df of the “residual” vectors *Z* (HT2004) once reasonable estimates for *α* and *β* have been obtained.

The previous conditional model can be easily generalized to the *d* + 1 vector (**X**,*Y* ) by simply replacing the *X*, *a*(*Y* ), and *b*(*Y* ) in Equation (2) by vectors **X**, **a**(*Y* ), and **b**(*Y* ), respectively, which means that vectors of parameters (*α*_1_, …, *α*_*d*_) and (*β*_1_, …, *β*_*d*_) must be estimated, and *G* is a multivariate df for **Z** (cf. Heffernan and Resnick, 2007).

The proposed estimation method from HT2004 is semi-parametric and involves several steps:

1. Estimate the marginal dfs for each variable separately.
2. Transform each variable in order that they each follow a marginal standard Gumbel (or, e.g., Laplace) df.
3. Estimate the parameters of the parametric model conditional on large values of the conditioning variable.
4. Information about *G* (e.g., functionals such as the mean, variance, quantiles, etc.) can be simulated using the empirical df of the estimated standardized residuals. Back transformation can be used to put these estimates onto the original scale.

HT2004 suggest using a hybrid, semi-parametric, model for step 1 of the following form that accounts for both the extreme and non-extreme values (cf. Coles and Tawn, 1991, 1994).


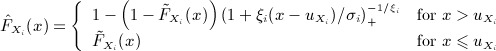
(5)

where 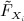 is the empirical df of the *X*_*i*_ values

In this study, we transform the variables using the Laplace transformation in step 2 following Keef *et al.*, 2013a, 2013b). HT2004 used non-linear least squares estimation on Equation (4) to estimate *α*_*i*_ and *β*_*i*_ for each *X*_*i*_ under a working assumption that *Z* follows a normal df. Of course, assuming a normal df for *Z* is incorrect as it implies that *X*_| *Y* > *u*_ is also normally distributed, which generally is not the case. To counteract the inherent estimation bias from this approach, Keef *et al.* (2013a) imposed joint constraints on the dependence parameters (*α*,*β*) in order to limit the upper quantiles of *X*_| *Y* > *u*_ to be less than or equal to *x*_*F*_, the value that would be observed under asymptotic dependence. From these estimates in step 3, HT2004 obtain new estimates 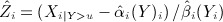 from which simulations from 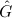 are obtained. In this step, it is important to keep the dependence structure inherent between the variates by keeping them together when performing the resampling of the residual vectors *Z*.

Uncertainty inference is carried out presently through bootstrap sampling in order to incorporate the uncertainty at each stage of the estimation procedure. We follow the strategy suggested by HT2004, the key component of which is the data generation step, which must be implemented carefully in order to maintain both the marginal and dependence features of the multivariate data. The original data are first transformed to X^*^ using the Laplace transformation using the estimated df described by Equation (5). In particular,


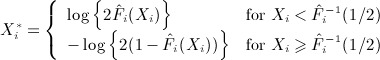


where 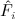 is estimated according to Equation (5) using MLE for the GP portion and empirical estimation for 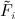. Next, a nonparametric bootstrap is obtained by sampling with replacement from the transformed data. The marginal values of this bootstrap sample are rearranged in order to preserve the associations between the ranked values in each component by replacing the ordered samples with ordered samples of the same size from the standardized Laplace df. The resulting sample is back transformed to the original marginal scales. In order to maintain relationships between the *X* variates, the nonparametric bootstrap samples are kept together. That is, for each variate *X*_*i*_, if the fourth entry for variate *i* is selected, then the fourth entry for all other *X*_*j*_ is also selected.

Model fit can be diagnosed by plotting the residual terms 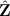 (as well as 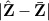) from the fitted model against the quantiles of the conditioning variable. A loess curve is also graphed. Any trend in these graphs suggests a violation in the assumption of independence between **Z** and the conditioning variable (cf. Equations (2) and (4)). Further diagnostics include plotting the original data on the original scale against the fitted quantiles of the conditional df. A good model fit should have good agreement in their quantiles conditioned on high values of the conditioning variable.

Note that this approach differs from that of incorporating covariates into the parameters of a univariate extreme value df in that a distribution for values of one variate is conditioned on only the extreme values of another variable. Therefore, the dependence is on the processes themselves rather than indirectly through distributional parameters.

The conditional EVA carried out in this study uses the R (R Development Core Team, 2012) package texmex (Southworth and Heffernan, 2011), which allows for the constrained estimation of the dependence parameters with the Laplace transformation on the marginal variables. For additional helpful references on the HT2004 conditional modeling approach, see Heffernan and Resnick (2007), Keef (2007), Keef *et al.* (2009a, 2009b, 2013a, 2013b); Jonathan *et al.* (2010, 2012); Lamb *et al.* (2010).

## 3. DATA

**Figure 2:**
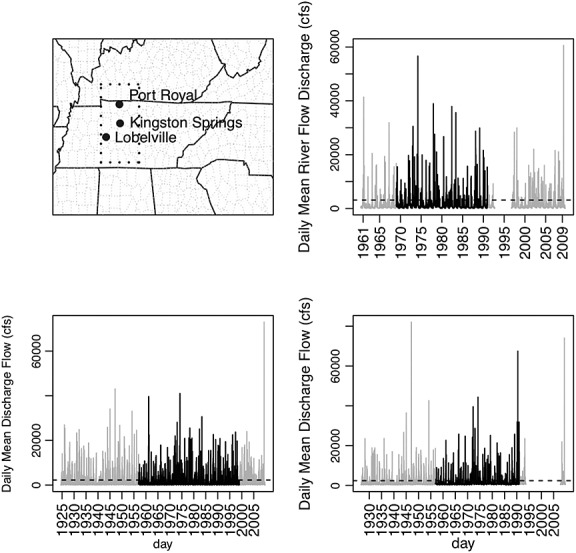
Top left: map of the three River measuring locations in Tennessee (at Port Royal and near Kingston Springs and Lobelville, respectively). Dotted rectangle shows the areal coverage of a reanalysis grid point (1.874 ^*o*^ longitude × 1.915 ^*o*^ latitude) if it were centered on Kingston Springs (Nashville is located at about the “K” in the Kingston Springs label). Daily mean stream flow discharge (cfs) is shown for the Red River at Port Royal, Tennessee (top right), Harpeth River near Kingston Springs, Tennessee (bottom left), and the Buffalo River near Lobelville, Tennessee (bottom right). Gray lines indicate the full available record, and black lines indicate the record available coincidentally with the WmSh reanalysis data used in the analyses in Section 4.2. Dashed horizontal lines are the 90th percentile for each river for data points shown in black

We use CAPE and Shear derived from a subset domain over North America from the global National Center for Atmospheric Research (NCAR)/United States National Center for Environmental Prediction reanalysis (see Kalnay *et al.*, 1996 and Brooks *et al.*, 2003 for more details). The original output employed here contained six hourly values every day for 42 years (1958–1999). Because the interest is in extremes, the daily maximum for each variable at each grid point is first taken to reduce the number of data points, which tend to occur at the same time every day, yielding a total of 15,300 non-missing data points at each grid location.† The spatial resolution of the reanalysis is 1.874 ^*o*^ in longitude by 1.915 ^*o*^ in latitude (see the dotted rectangle in Figure 2 top left panel), which corresponds to a 52 x 17 grid matrix with 884 grid cells over North America, each covering about 200 km ^2^.

Following previous studies (cf. Brooks *et al.*, 2003, 2007; Marsh *et al.*, 2007, 2009; Trapp *et al.* 2007, 2009, Heaton *et al.*, 2011), and based on reasonable discrimination of severe weather environments seen in Figure 1, and for the reasons explained in Section 1, values of CAPE have been converted to *W*_max_ (m s ^− 1^), and all analyses here are conducted for the product of *W*_max_ and Shear (m ^2^ s ^− 2^, henceforth referred to as WmSh). Note that while this product appears to discriminate severe weather environments fairly well (cf. Figure 1), it is possible for one of the variables to dominate the product so that both are not concurrently extreme. One might add complexity (see, e.g., Trapp *et al.*, 2009) in order to obtain a more reliable indicator. However, we note that even with such added complexity, an exact correspondence between high values and severe weather does not necessarily exist, and yet, in certain places, the occurrence of high values of just one of these variables can be important. Therefore, in the present work, we simply use WmSh without any additional requirements.

In order to investigate the full behavior of WmSh and how different processes can potentially contribute to the spatial extent of extreme weather, we stratify our dataset by four seasons defined here as: winter (DJF), spring (MAM), summer (JJA), and fall (SON).

This approach of segmenting the data can also be used to compare results for different periods. Here, we segment the data into three periods (1958–1978, 1979–1993, and 1994–1999), reflecting important changes in the quality of input data used in the reanalysis. The final short segment covers a period over which changes in large-scale climate have become quite noticeable. However, any other segmentation is possible, and future work will investigate how the spatio-temporal structures have changed and will change in the future based on climate model projections.

To explore connections on the impact level, we also investigate daily mean stream flow discharge (cfs) from three rivers in Tennessee (Figure 2): The Red River at Port Royal (top right), the Harpeth River near Kingston Springs, (bottom left), and the Buffalo River near Lobelville (bottom right).

All of the stream flow discharge data were obtained from the US Geological Survey online database (http://waterdata.usgs.gov), the most recent of which are considered provisional and subject to revision. The full record for the Red River is from 1 August 1961 through 28 September 2010. Data for Harpeth River near Kingston Springs run from 1 August 1925 through 28 September 2010, and for Buffalo River, they run from 1 October 1927 through 28 September 2010. All data were retrieved on 29 September 2010. They largely overlap with the reanalyis data (with the Red River series exhibiting some missing values in the common years).

## 4. RESULTS

The EVA approach of HT2004 is well suited to handle the data properties considered here: in particular, WmSh shares some properties in common with precipitation fields in that there are often many grid points and days with zero WmSh. Further, some grid points tend to have considerably larger values of WmSh than others (e.g., over the central USA vs. the northwest Pacific Ocean). The conditional EVA approach allows for these discrepancies among the different spatial locations because their entire distributions are modeled (not just the extremes).

To confirm appropriateness of the assumptions of independence between **Z** and q75 (when q75 is large) and the appropriateness of the underlying model, different diagnostic plots for several locations were consulted (not shown). Evaluations of these diagnostics suggest the appropriateness of the model fit (e.g., see Figure 4).

### 4.1. Spatial extremes for WmSh from the National Center for Atmospheric Research/United States National Center for Environmental Prediction reanalysis for North America

Conditioning requires a variable, which one could consider as varying over space (e.g., for a location conditioned on all sites within a fixed radius, similar to the analysis in Keef *et al.*, 2009a). However, this is not necessary here because our interest is in the overall evolution of severe weather spatially over time. For the present study, therefore, we consider a more general conditioning variable that summarizes the amount of WmSh energy over space for the entire field at each time point. Different options for such a measure of the energy could include, for example, an overall sum, a field average, or a high quantile of WmSh. Here, we choose to condition on the upper quartile of the WmSh energy across all grid values at each time point, henceforth q75 (m ^2^ s ^− 2^). The result is a univariate time series that captures a balanced summary of the intensity of the larger values of WmSh over space at each time point.

We also investigated other quantiles and overall integrated field measures (sum, average) as measures of field energy. Briefly, the field sum naturally yields higher values, but otherwise, spatial patterns and properties tend to mimic those for q75. For the present study, we focus on the method rather than the specific choice of field-energy summaries.

Note that by conditioning on extreme values of a measure of field energy (in this case q75) using the HT2004 model, we are able to ascertain the spatial cohesiveness, locations, and patterns of the WmSh field at time points when WmSh is intense typically over one or more sub-regions. Such an approach differs considerably from previous extreme value analyses for such indicators. For example, Heaton *et al.* (2011) investigate return level atlases obtained from an imposed spatial distribution on the parameters of extreme value distributions at individual grid points through a BHM, an approach that is useful for determining local risk for severe weather environments. The present analysis allows for a study of the spatial structure and its physical properties of WmSh when severe storm environments exist.

It is important to understand that conditioning on extreme values of q75 can ignore important high values of WmSh if they are restricted to limited areas. One way to account for such bias is to repeat the study for smaller regions or different time intervals, such as different decades, or as in this study, seasons. Such stratifications enable a more thorough analysis of underlying changes in intensity, spatial cohesiveness, spread, and the relationships with other large-scale processes. However, the focus, here, is on the most extreme activity over time, and other analyses may be necessary to fully capture all of the pertinent behavior of WmSh as it relates to severe weather environments.

**Figure 3:**
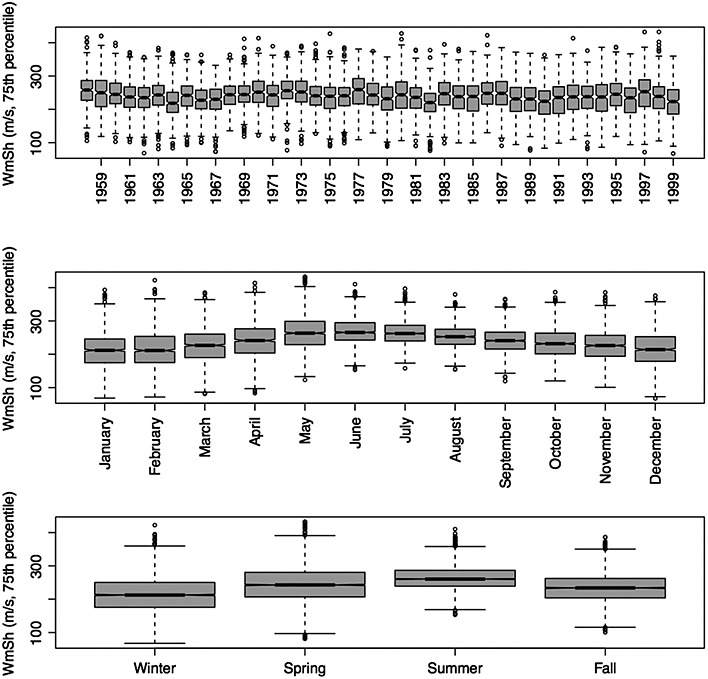
Box plots for the 75th percentile taken over the spatial field for WmSh (m s ^− 1^) for the 15,300 days (i.e., q75) stratified by: year (top), month (middle), and season (winter DJF, spring MAM, summer, JJA, and fall SON, bottom)

Figure 3 shows summaries of the annual distribution of q75. Although variability is evident (top), no obvious long term trend exists in these data. A marked seasonality (middle and bottom) is apparent, however, where the peak field energy clearly occurs in the spring and summer months. This observation agrees with the availability of convective energy that is maximized when cool air from mid-latitudes flows across increasingly hot and humid air from the south: a maximization of vertical instability (*W*_max_) and significant Shear toward summer.

The first step of the conditional EVA method, after deciding on a variable on which to condition the analyses, is to fit marginal dfs to each variable in order to apply the Laplace transformation. To do so, a hybrid of a GP df for values above a high threshold and the empirical df for values below the threshold is fit for q75 and WmSh (cf. Reiss and Thomas 2007; MacDonald *et al.*, 2011) separately at each grid point. It is found that the 90th percentile results in good fit diagnostics (not shown) for q75 and arbitrarily selected locations.

Our key interest lies in the spatial distribution of WmSh given high field energy (i.e., q75 larger than its 90th percentile over time), which we obtain from this procedure but only through simulations. That is, we do not obtain a closed-form parametric df, but simulated realizations from it. To investigate its properties, such as the mean and low and high quantiles, we graph the associated values spatially. Given that our interest is in extremes, it may at first appear strange to focus on low quantiles or even the mean of this df. However, because the df is conditional on high values of q75, such maps are informative of, in the case of low quantiles, a best-case scenario, or in the case of the mean, the expected spatial patterns and locations of high WmSh when it is extremely high over a large subregion of the domain. Of course, it would also be possible to map the spatial distribution of WmSh given a specific high return level of q75, which would give a type of “flood atlas” for WmSh that preserves physically realistic behavior.

**Figure 4:**
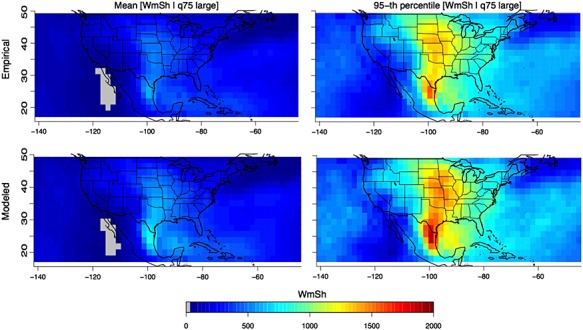
Top row: mean (left) and 95th percentile (right) of observed WmSh (m ^2^ s ^− 2^) when the 75th percentile taken over the spatial field for each of the 15,300 days (q75) is larger than the 90th percentile of q75 calculated over the entire 42-year record. Bottom row: mean (left) and 95th percentile (right) of WmSh (m ^2^ s ^− 2^) from the simulated distribution of WmSh conditional on high values of q75. This figure is available in color online at http://wileyonlinelibrary.com/journal/environmetrics

The top row of Figure 4 shows the mean (top left) and 95th percentile (top right) of observed WmSh (m ^2^ s ^− 2^) when q75 is larger than its 90th percentile over the entire record for comparison with results from the conditional EVA distribution shown in the bottom row of the figure: mean (bottom left) and 95th percentile (bottom right) of WmSh from the simulated conditional df for *q*75 > *u*, where *u* is the 90th percentile of q75 over the entire time record. Clearly, the model characterizes the observed behavior well. The advantage to having a valid model for WmSh conditioned on the extremes of q75 (or other field-energy measure) is that one can now make conjectures about field behavior under extreme return levels of q75; values that may not have been observed, but are reasonable to expect, as well as having a framework from which hypothesis testing can be conducted.

Note that the values in the bottom row of Figure 4 are generally considerably more extreme than those in the top row, and that they are maxima taken separately so that dependence is modeled over variables occurring at different time points. This illustrates a fundamental difference in approaches between the conditional approach adopted here and other spatial EVA models that utilize multivariate extreme value dfs or univariate extreme value dfs with spatially varying parameters. The conditional EVA model employed here demonstrates how the spatial WmSh process behaves when an extreme amount of energy exists over a relatively large portion of the spatial domain, even if many of the grid points are not at all extreme.

**Figure 5:**
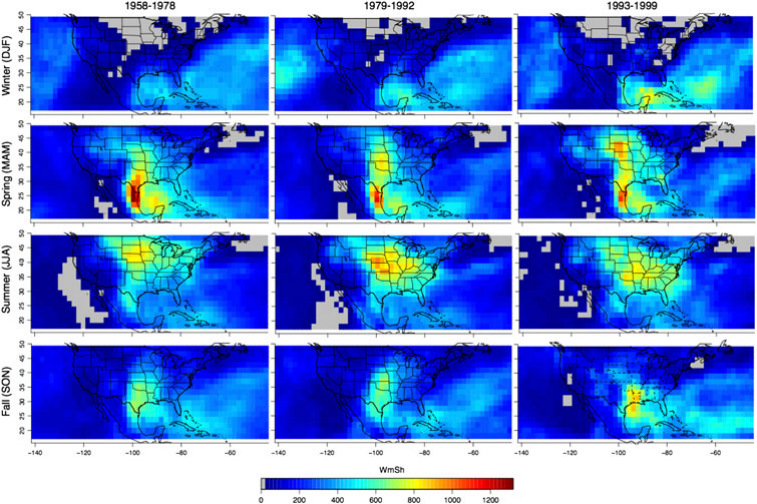
Mean simulated WmSh (m ^2^ s ^− 2^) conditional on high values of the 75th percentile taken over the spatial field of WmSh for each day in winter (top row), spring (second row), summer (third row), and fall (bottom row). High values are defined here as being above the 90th percentile taken over the season for 1958–1978 (left column), 1979–1992 (middle column), and 1993–1999 (last column). This figure is available in color online at http://wileyonlinelibrary.com/journal/environmetrics

Figure 5 shows similar graphs as the bottom row of Figure 4, but models are fit to data that are separated by season and for different time periods as described in Section 3. Winter results (top row) show that WmSh is increasing considerably over time in the southern region of the Gulf of Mexico, Yucatán Peninsula, and east of the Bahamas in the Atlantic Ocean. The mean simulated WmSh for this region may be associated with increased sea surface temperatures in the most recent 5 years of the data record for the winter season. A clearly defined area of high WmSh that is particularly apparent in period 2 (top right) exists in the southern North Pacific to the western coast of the USA, possibly associated with El Niño activity.

Not surprisingly, the Spring season (Figure 5, second row) is characterized by the emergence of thunderstorm activity in a clear band of extreme simulated WmSh stretching from about Nebraska and Iowa down to eastern Mexico and the central Gulf of Mexico, with the most intense values hovering over the eastern coast of Mexico. The upper branch of this overall region is sometimes referred to as tornado alley because it is a distinct region with frequent heavy thunderstorms and tornados in the spring and summer months. The large values on the east coast of Mexico are attributed to heating over dry land in this season that is associated with high CAPE (not shown). Further north, the values also reflect the still strong shear from westerlies aloft, which represent the cold-season conditions that taper out (or retract northward) as the summer progresses.

Similar activity is found in Figure 5 for the summer (third row), but noticeably lower WmSh is simulated south of the USA than in the spring months. The slim band of high simulated values in the central USA in the spring is considerably wider in summer, covering most of the plains and midwestern states in period 1 and stretching through to the southeastern states as well in the later periods. The most intense predictions, however, occur during period 2.

Results for fall are shown in Figure 5 (bottom row), where a strong increase in simulated WmSh in period 3 over the first two periods is evident, particularly around the eastern border of Texas and the western border of Louisiana. WmSh predictions also increase in the Gulf of Mexico and especially stretching south from this region out into the Atlantic Ocean for period 3.

Of course, the most important question to be addressed from these analyses concerns how WmSh is changing over time. Is it becoming more intense in critical areas? Are high intensity values migrating in space? For the present study, these questions are addressed by investigating differences between the periods of interest.

**Figure 6:**
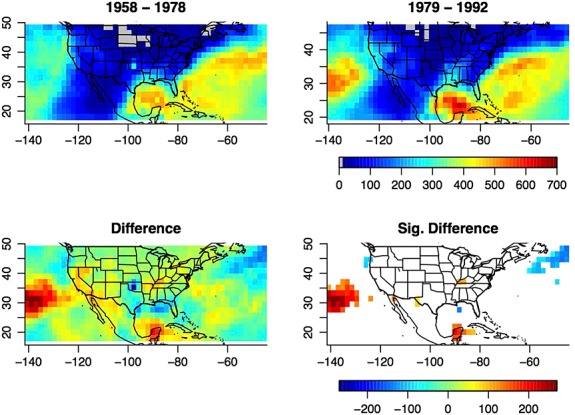
The top two panels are the same as the top row first two columns in Figure 5 but with a scale representing the range of data only for winter (rather than for all seasons). Bottom left panel shows the differences in mean simulated WmSh (m ^2^ s ^− 2^) conditional on the 75th percentile of WmSh over space being large over time between the first two time periods (i.e., top right panel minus top left panel). Bottom right panel is the same but only differences found to be statistically significant (based on bootstrapping) are displayed. This figure is available in color online at http://wileyonlinelibrary.com/journal/environmetrics

Figure 6 shows results for the differences in the mean simulated WmSh based on this conditional EVA model for the winter season. The top two panels are the same as the top row and first two columns in Figure 5 but with a scale particular to only the first two winter periods. The bottom two panels show the difference between the two means. The bottom right panel shows only the values found to be statistically significant at the 10% level or better. The significance test is made using a normal approximation interval with the variance obtained from the bootstrap procedure. What can be gleaned from the differences is that the majority of the region is not showing statistically significant changes in WmSh for winter from the first to second periods. Although spatial correlation is taken into account in the significance testing, no attempt has been made to account for multiple testing issues, which could be handled, for example, using a false discovery method (e.g., Benjamini and Hochberg, 1995; Ventura *et al.*, 2004; Benjamini and Heller, 2007). As only about 8% of the grid points show significant differences, multiple testing issues are a concern here and should be considered before making strong conclusions about changes in WmSh between these periods.

A few areas have grid points with lower WmSh activity, such as the Oregon coast and the North Atlantic just east of the Canadian and New England coast, as well as one grid point in the Gulf of Mexico due south of the eastern most part of Louisiana. A couple of reasonably large areas have numerous grid points showing statistically significant increases (by over 200 m ^2^ s ^− 2^) of WmSh: namely, over the southern North Pacific Ocean and over the Yucatán Peninsula. Although WmSh intensities in the southern North Pacific Ocean decline in the most recent period, an intensification occurs over the Yucatán Peninsula. These large-scale structures could be associated with variations in regional modes of variability typical to the climate system (e.g., El Niño, La Niña, Atlantic Multidecadal Oscillations, Pacific Decadal Oscillations, etc.).

**Figure 7:**
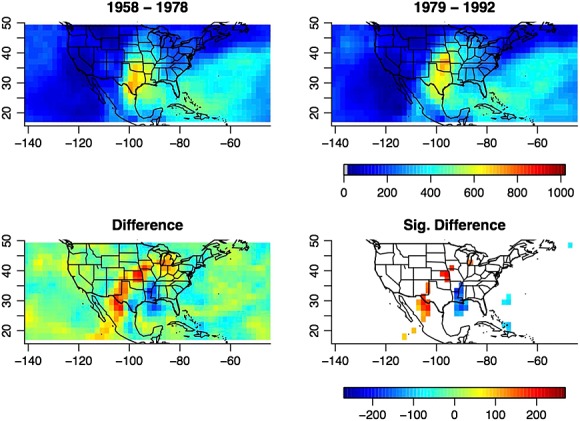
Same as Figure 6 but for fall instead of winter. This figure is available in color online at http://wileyonlinelibrary.com/journal/environmetrics

Differences for the fall (Figure 7) indicate a band of differences from the first to second period of increasing WmSh ( > 100 m ^2^ s ^− 2^) stretching up from southwest Mexico through the state of Chihuahua and eastern Texas, through eastern Kansas and northeastern Oklahoma, then up to northern Missouri, and finishing in the Great Lakes area. When accounting for statistically significant changes, this band is still apparent, only thinner. Close inspection of the upper two panels in the figure suggests that these changes may mostly be the result of a change in the location of severe WmSh activity, rather than an intensification. One could check this by first applying a technique such as image warping (e.g., Hoffman *et al.*, 1995; Alexander *et al.*, 1998, 1999; Nehrkorn *et al.*, 2003; Aberg *et al.*, 2005; Gilleland *et al.*, 2010b, 2010a) in order to quantitatively account for these possibilities (such analyses are beyond the scope of the present work). A smaller band of decreasing WmSh (on the order of 100 to 200 m ^2^ s ^− 2^ lower) is present over the western southeastern USA and in the Gulf of Mexico south of Louisiana, with statistically significant grid points over most of Louisiana and Mississippi, stretching into the Gulf. Together, they indicate a westward shift of the severe weather activity. Again, only about 4% of the grid points show significant differences, so multiple testing issues should be considered before drawing any conclusions.

An observation that can be made about the use of q75 as a conditioning variable within the conditional EVA approach is that lower WmSh predictions occur outside the regions where the bulk of the energy is apparent. That is, for the average simulated values from the conditional model over the entire record (Figure 4, bottom row), WmSh is very high in the tornado alley and hurricane prone regions, as well as slightly higher in the southern North Pacific Ocean. However, in other areas, the predictions are much lower (e.g., it is zero on average over most of Baja California, extending directly south for hundreds of miles). Similarly, in the winter, large regions of zero WmSh are simulated on average for most of the northern USA, as well as for pockets of regions in other areas depending on the period. In spring, the coastal areas of the northern Atlantic and again Baja California and extending southward, show average WmSh simulated to be essentially zero. These same areas exhibit values considerably larger for the summer, except for the most recent period. Fall generally has lower WmSh, but large areas of zero WmSh are simulated in the most recent period. In comparison with Figures 4 and 5 in Heaton *et al.* (2011), which show only what is happening in the extremes at each grid point, this observation from the conditional analyses demonstrates an advantage of the approach in that it enables predictions of non-extreme values in addition to the extremes. A more physically meaningful picture of WmSh is achieved from this perspective than from simply investigating the marginal GP df fits, or from the spatial EVA approaches that implicitly assume simultaneously extreme events. At the same time, the method provides a mechanism for making projections of probabilities for extreme events taking into account the spatial structure of the field.

### 4.2. The impact level

Because WmSh is a large-scale indicator for severe weather, we investigate how extreme events may be related to WmSh at an impact-level variable; that is, a variable that occurs at a fine spatial scale where their extremes can have a major impact on society (e.g., tornados, river flow, etc.). To that end, WmSh is modeled conditional on large values of stream flow from three rivers in northeast Tennessee. While it may seem strange to condition on high stream flow, rather than the other way around, we note that we are after the spatial distribution of WmSh over the region associated with such high stream flow episodes. It is these patterns that are of interest.

We choose Tennessee because of a heavy precipitation event that occurred in that area in 2010 and resulted in a devastating flood in Nashville, Tennessee (see, e.g., Durkee *et al.*, 2011). According to the National Weather Service Hydrometeorological Design Studies Center (http://nws.noaa.gov/oh/hdsc/index.html), the estimated precipitation over large portions of Tennessee represented roughly a 1000-year event (Durkee *et al.* 2011). Although our data record does not cover this exceptional event in 2010, it is of interest to investigate the types of spatial patterns that relate WmSh to extreme river flows in this region, and if these analyses would yield similar results as the conditions that actually occurred in spring 2010. In conjunction with heavy precipitation, the events, which occurred from 1–2 May 2010, included a series of strong thunderstorms with reports of 41 tornados, 57 severe winds, and 43 severe hail episodes, conditions generally closely associated with high WmSh (see previous texts).

For each river, a threshold equal to the 99th percentile of river flow data is found to yield the best fit diagnostics (not shown) for fitting the marginal GP df (the 90th percentile resulted in very poor diagnostics). The 90th percentile of WmSh is used as a selection of fit diagnostics (not shown) revealed that this value is adequate for the threshold for this variable. For the subsequent conditional EVA fits (step 2 of the HT2004 estimation method), the 99th percentile is used. Selected plots of *Z* from Equation (4) against high quantiles of the conditioning variables do not show any obvious trends indicating that the threshold choice is appropriate.

For the Red River at Port Royal, Tennessee, we analyze a subset of the data that are available for the same days as the WmSh reanalysis product (black lines in Figure 2). Scatter plots (not shown) of WmSh against daily mean discharge flow for the Red River at Port Royal, Tennessee for nearby reanalysis grid points do not reveal any obvious association between the variables. Nevertheless, because of the rarity of extremes, by definition, it is of interest to determine if there is any dependence between the two when at least one of the two variables is extreme.

To gain an understanding of possible associations in their extremes, we concentrate on a couple of grid points from the WmSh reanalysis that are nearby the river locations. When the conditional EVA model is fit to WmSh conditional on high stream flow at Port Royal, the mean simulated value is on the order of 280 m ^2^ s ^− 2^, whereas the nearest grid point to the other two locations of this study shows a mean simulated WmSh on the order of 370 m ^2^ s ^− 2^ for all seasons. The conditional value of WmSh is greatest for the spring.

**Figure 8:**
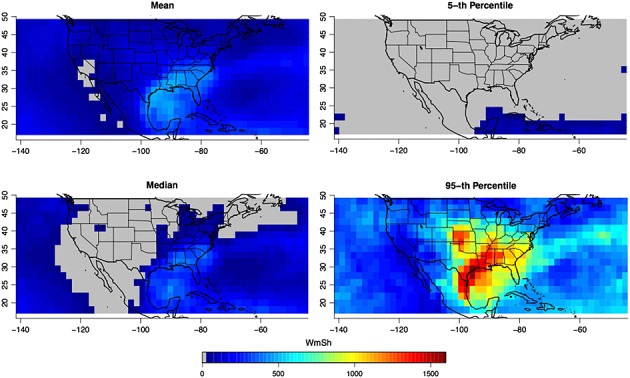
Predicted WmSh (m ^2^ s ^− 2^) conditional on high values of stream flow from the Red River in Tennessee shown by the black lines in Figure 2. Top left panel is the simulated mean WmSh, top right is the simulated 5th percentile WmSh, lower left is the median simulated WmSh, and lower right is the 95th percentile of simulated WmSh. This figure is available in color online at http://wileyonlinelibrary.com/journal/environmetrics

Previous results from the conditional modeling approach highlight an advantage of the method over other spatial EV methods in that more complicated relationships between the extremes of variables can be identified and then estimated. In particular, for WmSh conditioned on high values of river flow in the selected Tennessee Rivers, physical characteristics of high WmSh advection from the south are evident coincidentally with high river flow in this region, whereas WmSh is considerably less extreme nearly everywhere else (cf. Figure 8).

Figure 8 displays results of simulated WmSh given that the stream flow discharge is larger than its 99th percentile for the Red River at Port Royal, Tennessee. High simulated values of WmSh are evident near the measuring location of the river (Figure 2 top left) and to the south. The 5th percentile of the simulations is zero for most of the domain, but relatively larger values exist to the south over the Yucatán Penninsula, north into the Gulf of Mexico, and east into the Atlantic Ocean. The distribution of WmSh conditional on high values of river flow is characteristic of advection from the south but also demonstrates that the dependence between the two variables is relatively weak. Results at the other two rivers (not shown) are analogous to the Red River. These analyses were also carried out by season, and similar results (not shown) are obtained, though it is clear that the strongest associations are evident in the spring and summer seasons.

**Figure 9:**
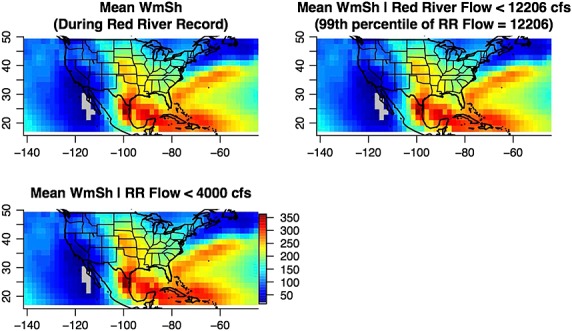
Average values of WmSh (m ^2^ s ^− 2^) conditioned on river flow (cfs) from the Red River in Tennessee. Top left is for all values of river flow, top right is the average conditional on river flow less than its 99th percentile, and bottom left is conditioned on river flow less than 4000 cfs. This figure is available in color online at http://wileyonlinelibrary.com/journal/environmetrics

Of course, as pointed out by a reviewer, it would be more interesting to study how good of a predictor the spatial patterns of WmSh are for extreme river flow. A full analyses of such predictability is beyond the scope of the present treatment. However, an important consideration concerns the prevalent values of WmSh. Figure 9 shows empirical averages for WmSh during the record for the Red River in Tennessee, as well as under lower river flows. It can be seen that while the advection from the Gulf of Mexico is prevalent, it is “aimed” further west than when high stream flow occurs when it is “aimed” more directly at the river catchment area (cf. Figures 8 and 9). Further, considerable WmSh prevails elsewhere, where it typically does not when river flow is extreme.

## 5. SUMMARY, DISCUSSION, AND CONCLUSIONS

The conditional EVA approach introduced by Heffernan and Tawn (2004, HT2004) is applied to a large-scale indicator for severe weather, namely, the product of the vertical instability indicator *W*_max_ and 0–6 km wind shear (WmSh) reanalysis data over North America by conditioning on large values of its 75th percentile across the grid at each time point. It is found that physically meaningful patterns are discerned from the approach making it a useful new tool for analyzing extremes under a changing climate.

We demonstrate how this type of analysis can be used to make inferences about future extremes when the character of the spatial processes is complex. For example, WmSh may not be extreme or changing in many regions while it is extreme and/or changing in other regions. Most previous spatial extreme analyses require simultaneous extremes to be valid; otherwise, the dependence is over extremes that occur at possibly different times of the year, etc.; that is, most models for spatial extremes are suitable for asymptotic dependence. Some recent exceptions include Wadsworth and Tawn (2012) and Davison *et al.* (2013). Some more recent work on multivariate GP distributions (Rootzén and Tajvidi, 2006) may also help to resolve this issue. The conditional approach taken here allows for studying the entire spatial distribution of WmSh under severe storm environments, and spatial correlation is taken into account in the significance testing; although not multiple testing issues, which could be handled, for example, using a false discovery method (e.g., Benjamini and Hochberg, 1995; Ventura *et al.*, 2004; Benjamini and Heller *et al.*, 2007).

The analysis here does not account for temporal dependence or structure, which may be important when making inferences for WmSh as a severe weather indicator. Such dependence could be accounted for by imposing covariates on the parameters in Equation (3; Keef *et al.*, 2009b, or possibly by implementing an alternative bootstrap algorithm that accounts for temporal dependence (e.g., Lahiri, 2003), or by declustering extremes of the conditioning variable (e.g., Fawcett and Walshaw, 2007). One drawback to the conditional approach is the nature in which the model needs to be fitted to the data. It is a complicated semi-parametric process involving a mixture of maximum-likelihood estimation, non-linear least squares estimation, and pseudo-likelihood estimation (one of the greatest early criticisms of the approach; see comments to HT2004). Further, the procedure itself can be relatively computationally expensive and unstable for some data.

Ultimately, it may be of interest to investigate the uncertainty in the spatial distribution of WmSh conditioned on high field energy for particular return levels of the high field energy variable (e.g., q75 used here). A particular challenge for such analysis concerns the fact that WmSh may or may not be extreme at any given point. Therefore, it is not clear how best to portray such uncertainty. One possibility might be to investigate plots of the variance at each grid point.

Another potential application of the present statistical modeling scheme is in evaluating the ability of current climate models to reproduce the observed spatio-temporal structures of WmSh. One question of considerable interest is whether or not, from an ensemble of different models and multiple simulations, one can capture the dynamics properly, or if one should weigh some models more heavily than others (Tebaldi *et al.* 2004; Cayan *et al.* 2010; Mote *et al.* 2011). The same issue of how to utilize the uncertainty information provided by the bootstrap procedure will apply. One possible method might be to employ the spatial prediction comparison test introduced by Hering and Genton (2011) on simulated functionals of the df *G*, perhaps with the image warp loss function proposed by Gilleland (2013) to help distinguish between location and intensity errors. One could conduct the test for equal means (of the loss differential field), over each bootstrap sample to derive a sample of test statistics along with information about whether they are statistically significantly different from zero or not. Such a scheme may not provide a rigorous argument for weighting climate models in an ensemble but could present an improvement over current methods, especially as regards extreme behavior.

Application of the approach to river flow data demonstrates a complicated association between high values of WmSh and high values of river flow; one that cannot be readily discerned from multivariate EVA alone. The beauty of this approach lies in the ability to discriminate physically meaningful spatial structures of WmSh conditional on high river flows. As a reviewer suggests, using this method for prediction of probabilities of extremes would be difficult, here, because of the use of an 884-dimensional empirical distribution.

## Footnotes

† Some data are missing in the reanalysis. In particular, 6 days in 1978 (1–6 March), three in 1981 (1–3 March), nearly a month (28 days) in 1982 (3–30 November), 2 days in 1983 (30 and 31 October), and 1 day in 1985 (31 October).
